# Draft Genome Sequence of Putative 2-Methylisoborneol-Producing *Pseudanabaena yagii* Strain GIHE-NHR1, Isolated from the North Han River in South Korea

**DOI:** 10.1128/MRA.00431-20

**Published:** 2020-07-02

**Authors:** Ju-Yong Jeong, Sang-Hoon Lee, Mi-Ra Yun, Seung-Eun Oh, Tae-Hwa Kim, Mi-Hye Yoon, Hee-Deung Park

**Affiliations:** a Department of Water Environment Research, Gyeonggi-do Institute of Health and Environment, Suwon, South Korea; b School of Civil, Environmental, and Architectural Engineering, Korea University, Seoul, South Korea; University of Southern California

## Abstract

The draft genome sequence of *Pseudanabaena yagii* GIHE-NHR1, a filamentous cyanobacterium, is reported here. Comparative genome analysis suggests that this strain can produce an odor-causing compound (2-methylisoborneol) in water. The genome information is expected to improve the understanding of the putative 2-methylisoborneol production by the bacterium.

## ANNOUNCEMENT

The compound 2-methylisoborneol (2-MIB) generates earthy-musty odors in water and is known to be produced mainly by cyanobacteria, including those in the genus *Pseudanabaena* (1, 2). Pseudanabaena yagii is a filamentous cyanobacterium reported to produce 2-MIB (3). In November 2018, a 2-MIB odor occurred in the North Han River, which is used as one of the main water sources in the metropolitan area of Seoul, South Korea.

The influent of a water treatment plant in the upstream region of the North Han River was collected using a grab sampling method (4). Strain GIHE-NHR1 was isolated using a micropipetting method (5) and cultured in BG-11 medium (6) at 25°C under the conditions of a 16-h light/8-h dark cycle, according to an isolation method described previously (7). The genomic DNA was extracted using the DNeasy PowerSoil kit (Qiagen, Valencia, CA, USA) and was sequenced using the PacBio RS II platform and the Illumina HiSeq 2500 platform. The PacBio and Illumina sequencing libraries were prepared using the P6 PacBio DNA/polymerase binding kit, PacBio DNA sequencing kit v4.0, and SMRTCell 8M and the TruSeq library kit, respectively, according to the manufacturers’ protocols. After the removal of adaptors and low-quality sequences by self-error correction during the preassembly step using FALCON-integrate v2.1.4 software (https://github.com/PacificBiosciences/FALCON-integrate) (8), a total of 143,959 subreads (1,438,601,723 bp) were generated from the PacBio platform, with an *N*_50_ value of 13,782 bp, and used for the assembly step with the same software. From Illumina paired-end sequencing, 21,032,908 reads (average length, 100 ± 0 bp) were retrieved and used for error correction. The quality-filtered sequence reads, in which 90% of bases had a Phred score of ≥30, were used for error correction using Pilon v1.21 software (https://github.com/broadinstitute/pilon/wiki) (9). The final depth of the sequenced genome was 59×. The genome was annotated using the NCBI Prokaryotic Genome Annotation Pipeline (PGAP) v.4.11 (10, 11). Default parameters were used for all software unless otherwise specified.

From the PacBio sequencing, 9 uncircularized contigs with 5,567,508 total contig bases were retrieved. The *N*_50_ value of the contigs was 3,694,966 bp after the error-correcting assembly. Four of the nine contigs represented main chromosomes, and the other five contigs were expected to be plasmids in the BLAST search. The genome information provided from the PGAP showed that the genome contains 5,567,508 bp, with a G+C content of 42.3%, and 5,005 coding sequences, 55 tRNAs, 11 rRNAs, and 4 noncoding RNAs. Four CRISPR arrays were also discovered via the PGAP. Sequence analysis revealed that the 16S rRNA gene and 16S-23S rRNA internal transcribed spacer of strain GIHE-NHR1 showed 99% identity to those of a P. yagii strain isolated in Japan (3). Moreover, both geranyl diphosphate 2-*C*-methyltransferase and 2-MIB synthase, which are involved in the synthesis of 2-MIB, were found in the genome (12) and showed 100% identity to those of the strain from Japan. These findings suggest that strain GIHE-NHR1 is a 2-MIB-producing cyanobacterium. A gene cluster involved in nitrogen fixation (*nif*) was also discovered in the genome of strain GIHE-NHR1, although a heterocyst for nitrogen fixation was not observed in the microscopic observation.

### Data availability.

The draft genome sequence of Pseudanabaena yagii GIHE-NHR1 has been deposited in NCBI under accession numbers JAAVJL000000000 (GenBank), PRJNA616171 (BioProject), SAMN14483332 (BioSample), and SRR11458705 and SRR11458709 (SRA). Four contigs representing main chromosomes and five contigs representing plasmids have been deposited under accession numbers JAAVJL010000001 to JAAVJL010000004 and JAAVJL010000005 to JAAVJL010000009, respectively.
